# Spatiotemporal Characterizations of Dengue Virus in Mainland China: Insights into the Whole Genome from 1978 to 2011

**DOI:** 10.1371/journal.pone.0087630

**Published:** 2014-02-14

**Authors:** Hao Zhang, Yanru Zhang, Rifat Hamoudi, Guiyun Yan, Xiaoguang Chen, Yuanping Zhou

**Affiliations:** 1 Department of Infectious Diseases, Nanfang Hospital, Southern Medical University, Guangzhou, Guangdong Province, China; 2 Key Laboratory of Prevention and Control for Emerging Infectious Diseases of Guangdong Province, School of Public Health and Tropical Medicine, Southern Medical Guangzhou, Guangdong Province, China; 3 Department of Pathology, Rockefeller Building, University College London, London, United Kingdom; 4 UCL Cancer Institute, Paul O Gorman Building, University College London, London, United Kingdom; 5 Program in Public Health, University of California Irvine, Irvine, California, United States of America; University of Rochester, United States of America

## Abstract

Temporal-Spatial of dengue virus (DENV) analyses have been performed in previous epidemiological studies in mainland China, but few studies have examined the whole genome of the DENV. Herein, 40 whole genome sequences of DENVs isolated from mainland China were downloaded from GenBank. Phylogenetic analyses and evolutionary distances of the dengue serotypes 1 and 2 were calculated using 14 maximum likelihood trees created from individual genes and whole genome. Amino acid variations were also analyzed in the 40 sequences that included dengue serotypes 1, 2, 3 and 4, and they were grouped according to temporal and spatial differences. The results showed that none of the phylogenetic trees created from each individual gene were similar to the trees created using the complete genome and the evolutionary distances were variable with each individual gene. The number of amino acid variations was significantly different (*p* = 0.015) between DENV-1 and DENV-2 after 2001; seven mutations, the N290D, L402F and A473T mutations in the E gene region and the R101K, G105R, D340E and L349M mutations in the NS1 region of DENV-1, had significant substitutions, compared to the amino acids of DENV-2. Based on the spatial distribution using Guangzhou, including Foshan, as the indigenous area and the other regions as expanding areas, significant differences in the number of amino acid variations in the NS3 (*p* = 0.03) and NS1 (*p* = 0.024) regions and the NS2B (*p* = 0.016) and NS3 (*p* = 0.042) regions were found in DENV-1 and DENV-2. Recombination analysis showed no inter-serotype recombination events between the DENV-1 and DENV-2, while six and seven breakpoints were found in DENV-1 and DENV-2. Conclusively, the individual genes might not be suitable to analyze the evolution and selection pressure isolated in mainland China; the mutations in the amino acid residues in the E, NS1 and NS3 regions may play important roles in DENV-1 and DENV-2 epidemics.

## Introduction

Dengue is one of the most globally important vector-born infectious diseases in tropic and sub-tropic areas. It is caused by the single-stranded, positive-sense RNA, dengue virus (DENV) which is the member of the *Flavivirus* genus and the *Flaviviridae* family. DENV consists of four antigenically related serotypes (DENV-1, DENV-2, DENV-3 and DENV-4). The viral genome is approximately 11 kb in length and contains a single open reading frame (ORF) that encodes three structural proteins, including the capsid (C), premembrane/membrane (PrM/M), and envelope (E) proteins and seven non-structural (NS) proteins (NS1, NS2A, NS2B, NS3, NS4A, NS4B and NS5); this ORF is flanked by the 5′- and 3′-non-translated regions (5NTR/3NTR).

In mainland China, dengue fever cases have been reported every year since 1997, especially in the Guangdong province [1], [2], [3]. All four DENV serotypes have been epidemic. DENV-1 was responsible for dengue fever (DF) epidemics in Guangdong province in 1979 and 1985 [4]; several outbreaks caused by the same virus were also reported in 1991, and from 1995 to 2010 [1], [2], [5], [6]. DENV-2 caused DF epidemics in Hainan province in 1985, in Guangxi province in 1988, in Guangdong province in 1993, 1998 and 2001 and in Fujian province in 1999 [7]. DENV-3 has rarely caused epidemics in mainland China since 1982, and DENV-4 has always been sporadic and has consisted with other serotypes [7].

Co-circulation of multiple DENV serotypes, genotypes and clades in the same community has become common [8], [9], [10], [11]. At present, the most widely accepted method for genotyping DENV involves the phylogenetic analysis of gene sequences, in particular the E gene [12], [13]. Recent research has shown that individual genes, except the 5NTR gene, are suitable for genotyping DENV using the phylogenetic method in Thailand [14], a country in which dengue is seriously epidemic. The E gene of DENV has also been widely used in molecular and evolution analyses in mainland China [15], [16], [17]. Since the first documented DENV infection in Foshan in 1978, DENV has spread into mainland China during the last 30 years. However, there is a lack of research evaluaing whether the individual genes, including E gene, are suitable for genotyping the dengue viruses, as well as few analyses of their evolution and selection pressures. Recently, complete genome analysis of the West Nile virus and Japanese encephalitis virus, which belongs to the same family as DENV, has been shown to be a powerful tool for evaluating the relatedness and for reconstructing the evolutionary history and phylogeography of these viruses [18], [19], [20], [21], [22], [23], [24], [25], [26]. Spatial and temporal analyses of dengue fever cases in Guangdong province showed that the geographic range of the dengue fever epidemic has expanded during recent years [27]; counties around the Pearl River Delta area and the Chaoshan Region are at an increased risk for dengue fever [28]. Therefore, the characteristics of DENV epidemics in mainland China need to be described according to spatial and temporal analyses.

In this study, a total of 40 complete genome sequences of DENV (19 DENV-1; 11 DENV-2; 6 DENV-3; 4 DENV-4) were downloaded from GenBank and analyzed using bioinformatics methods. The two aims of this study were to determine whether individual genes are suitable for genotyping DENV and to characterize the molecular epidemiology and virology of the DENVs using the complete genome sequence by spatial and temporal analyses.

## Materials and Methods

### Virus

The complete sequences of dengue viruses were downloaded from GenBank (http://www.ncbi.nlm.nih.gov/genbank/). There were 19 DENV-1, 11 DENV-2, 6 DENV-3 and 4 DENV-4 viruses. The details of these viruses are shown in Table 1.

**Table 1:** The overall distance of DENV-1 and DENV-2 from individual genes.

Gene Area	DENV-1	DENV-2
	Mean ± Standard Deviation (M±SD)	Mean ± Standard Deviation (M±SD)
Whole genome	0.081±0.004	0.089±0.003
5′UTR	0.011±0.005	0.039±0.011
C	0.041±0.007	0.076±0.017
prM	0.055±0.009	0.098±0.022
E	0.084±0.009	0.079±0.008
NS1	0.091±0.015	0.089±0.011
NS2A	0.073±0.007	0.111±0.014
NS2B	0.094±0.02	0.099±0.021
NS3	0.082±0.009	0.092±0.009
NS4A	0.126±0.026	0.052±0.007
NS4B	0.073±0.011	0.092±0.012
NS5	0.080±0.007	0.072±0.006
3′UTR	0.035±0.007	0.017±0.006

### Genotyping Method

Phylogenetic analysis was performed on a gene-by-gene basis using the sequences of the coding region and the non-coding region of 40 DENV strains isolated in mainland China. Sequence alignments were performed using the Clustal W program, which resulted in alignments of the complete sequence and for each individual gene sequence for each of the four DENV serotypes. Maximum likelihood (ML) phylogenetic trees were then estimated using the MEGA (Molecular Evolutionary Genetics Analysis) 5.05 software. To determine the support for a particular grouping on the phylogenetic trees, bootstrap re-sampling analyses was performed using 1000 replicate neighbor-joining trees estimated by using the ML substitution model.

### The Evolutionary Distance of DENV using Individual Genes

The overall evolutionary distance of DENV using individual genes was determined using the Mega 5.05 software after the sequences were aligned. To determine the support for the distance calculating, bootstrap re-sampling analyses was performed using 1000 replicate neighbor-joining trees estimated using the ML substitution model.

### Variations of Amino Acids (AAs) in DENV-1, DENV-2, DENV-3 and DENV-4

The ORF gene was obtained by manually removing the 5NTR and 3NTR region. The number of amino acid changes was observed in the four dengue serotypes compared to each standard dengue strain (DENV-1: Hawaii, EU848545; DENV-2: New guinea-C, AF038403; DENV-3: H87, M93130; DENV-4: H241, AY947539). When a significant difference was observed in the equality of variances (p<0.1), the Kruskal-Wallis statistic was used to compare the number of AA variations among the four DENV-1, DENV-2, DENV-3 and DENV-4 groups, and Student’t t-test was used to compare DENV-1 and DENV-2 isolates in 2001–2010 group, when the data were not significant according to the normality and equality of variances (p>0.1). A one-way ANOVA test was used to compare the AA changes between the 17 DENV-1 and 8 DENV-2 viruses isolated during the past recent 20 years (1990–2010) because the data were not significant according to normality and equality of variances (p>0.1) analyses. According to the geography of these DENV-1 and DENV-2 isolates, the changes in AAs were also compared using the Kruskal-Wallis (significant differences in normality) or Student’t- t-test (non-significant differences in normality). All tests were two-sided, and a p<0.05 value was considered statistically significant. The differences in normality and equality of variances were considered significant when the p<0.1. This statistical analysis was performed using the SPSS software package (version 13.0).

### Recombination Analysis

All the DENV-1 and DENV-2 isolates were analyzed using “DataMonkey” (available online: http://www.datamonkey.org/). The recombination events of the dengue viruses were analyzed using the genetic algorithm (GARD). The GARD method for detecting recombination was demonstrated in a previous study [29]. The neighbor-joining trees between the breakpoints were also demonstrated.

## Results

To determine which gene is suitable for intra-serotype identification, phylogenetic analysis was performed separately using the sequences of the complete genome, the ORF region and each gene. Fourteen phylogenetic trees were generated using the ML method for the DENV-1 and DENV-2 serotypes (1 tree/1 gene). The bootstrap value was added to each major node. A bootstrap value is close to 100% at the nodes indicated, a more accurate genotype identification. A clade supported by a bootstrap value of at least 90% was considered highly significant. As a result of the small number of DENV-3 and DENV-4, these phylogenetic trees were not analyzed.

According to the different serotypes, location (Guangzhou and other regions) and isolation year, the AA variations were also determined to explain the DENV epidemic in mainland China using the ORF and individual genes.

### Genotyping DENV-1

**Figure 1:**
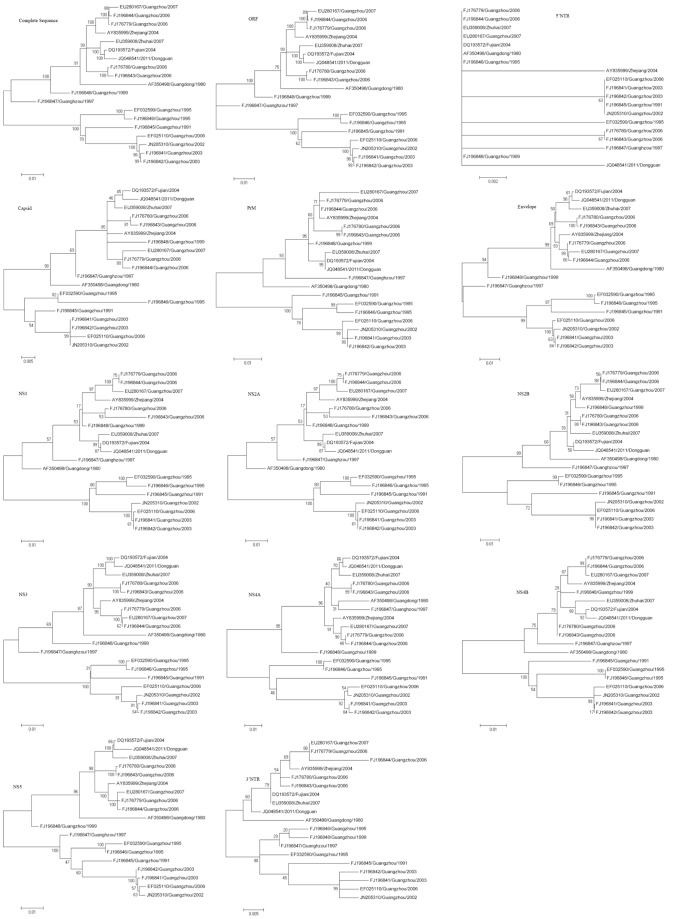
Phylogenetic analysis of DENV-1 as determined from 14 ML trees derived from the complete genome, ORF and individual gene sequence.

Phylogenetic analysis of DENV-1 included the creation of 14 ML trees derived from the complete genome sequence, the ORF and each gene (Figure 1). The ML trees generated from the ORF and the E region (1485 bp) were similar to the tree generated from the complete genome sequence; however, the trees generated from the ORF and the E gene were each supported by bootstrap values of less than 90% at some of the major nodes (Figure 1). The trees from other genes, including the 5′NTR (95 bp), C (342 bp), PrM (498 bp), NS1 (1056 bp), NS2A (654 bp), NS2B (390 bp), NS3 (1857 bp), NS4A (450 bp), NS4B (747 bp), NS5 (2706 bp) and 3′NTR (418 bp), were different from the tree generated using the complete sequence (Figure. 1). Therefore, all ML trees generated from the DENV-1 isolates showed different topology, suggesting that none of gene regions can be representatively used to describe the molecular characteristics of DENV-1 viruses in mainland China.

### Genotyping DENV-2

**Figure 2:**
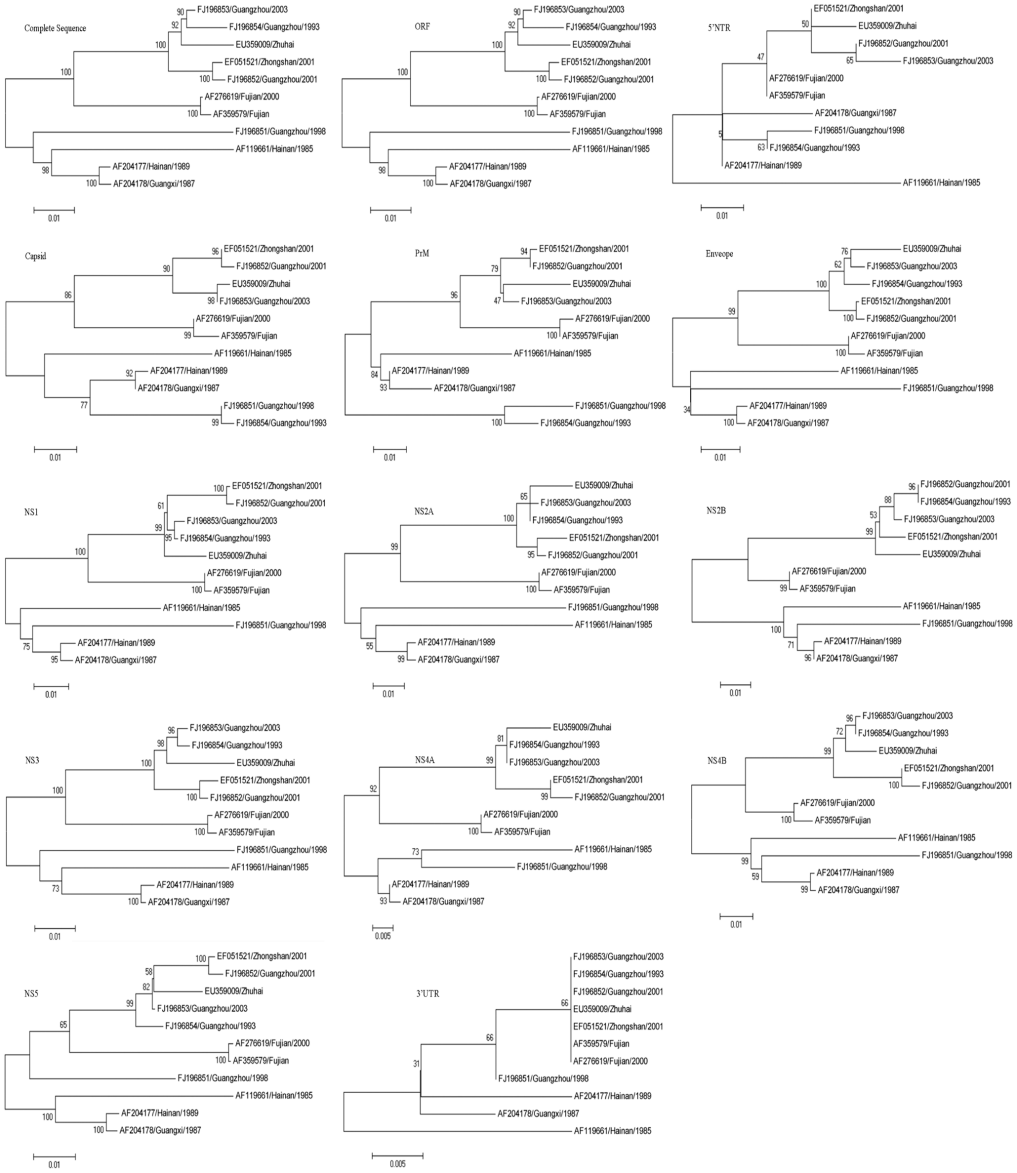
Phylogenetic analysis of DENV-2 as determined from 14 ML trees derived from the complete genome, ORF and individual gene sequence.

Phylogenetic analysis of DENV-2 included the creation of 14 ML trees derived from the complete, the ORF and each gene (Figure 2). The ML tree generated from the ORF was the same as the tree generated using the complete sequence, and the topological structure of the tree generated using the NS3 gene region was similar to the one generated using the complete sequence; however, the trees generated from the NS3 region were each supported by bootstrap values of less than 90% at the major nodes (Figure 2). The trees from each of the genes, including the 5′NTR, C, PrM, E, NS1, NS2A, NS2B, NS4A, NS4B, NS5 and 3′NTR genes, were different from the tree generated for the complete sequence (Figure 2), Therefore, all of the ML trees generated for DENV-2 showed different topology, which suggests none of these gene regions, except for the ORF, can be representatively used to describe the molecular characteristics of DENV-2 in mainland China.

### The Overall Evolutionary Distance of DENV

This analysis was performed for DENV-1 and DENV-2. The evolutionary distances were similar for the NS5 (0.080±0.007, 0.072±0.006), NS3 (0.082±0.009, 0.092±0.009) and NS1 (0.089±0.011, 0.089±0.011) regions for DENV-1 and DENV-2, compared to the distance of the whole genome (DENV-1∶0.081±0.004 and DENV-2∶0.089±0.003); the distance of the E gene was relatively far (DENV-1∶0.084±0.009 and DENV-2∶0.079±0.011), which demonstrates that the variability of the E gene. The evolutionary distances of other individual genes are detailed in Table 1.

### Amino Acid Sequence Variations in the DENV-1, DENV-2, DENV-3 and DENV-4 Viruses

**Figure 3:**
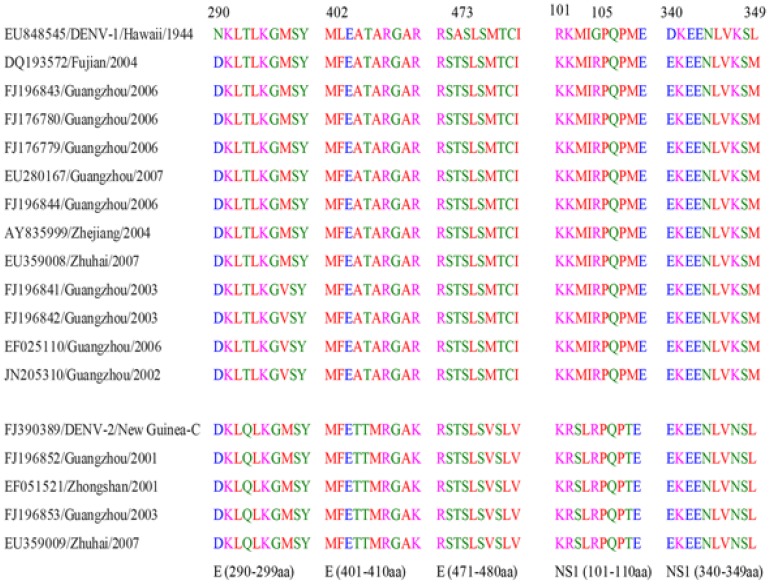
Special mutations in the E and NS1 gene regions of the DENV-1 isolates collected after 2000, compared with DENV-2.

The AA changes were significantly different in the ORF region of the four DENV groups (χ^2^ = 14.8, p = 0.002, Table 2). There were more changes in the AAs in the DENV-1 group (mean rank: 27), followed by DENV-4 group (mean rank: 22). As for the four groups based on the serotypes (DENV-1 and DENV-2) and the isolation year, a significant difference was shown in these four groups (F = 3.9, p = 0.024), while there was a significant difference in the AA changes between the two groups of the DENV-1 and DENV-2 strains isolated from 2001–2010 (p = 0.022, 89.2±14.2 *vs.* 64±6.7, Table 3). The AA changes in the E (Z = 2.96, p = 0.003) and NS1 (F = 0.4, p = 0.006) genes were significantly different between the DENV-1 and DENV-2 isolates from 2001–2010, as shown by the AA variations between these two genes (M±SD: 16.8±2.4 *vs.* 11±0.8; 15.3±3.0 *vs.* 7.5±1.7, respectively). According to the alignment of these DENV-1 and DENV-2 isolates, the N290D, L402F and A473T mutations in the E gene and the R101K, G105R, D340E and L349M mutations in the NS1 gene region of the DENV-1 isolates may be significant, while these AAs in the DENV-2 isolates have not been changed (Figure 3). The AA changes were more numerous in the viruses from Guangzhou, including the first reported dengue case in Foshan, than in the viruses from dengue epidemics outside of Guangzhou (NS1 of DENV-3: t = 2.3, p = 0.034; NS1, NS2B and NS3 of DENV-2: t = 2.7, 2.9, Z = 2.2; p = 0.024, 0.016, 0.042, Table 4).

**Table 2:** Comparison of the AA variations in the complete sequences of isolates from the four serotypes in mainland China.

Dengue serotype	Strains (accession number/geography/year)	AA variations, Mean±Standard Deviation (M±SD)	Kruskal-Wallis Test
DENV-1	EF032590/Guangzhou/1995	85±3	χ^2^ = 14.8
	FJ196846/Guangzhou/1995		p = 0.002
	FJ196848/Guangzhou/1999		
	JN205310/Guangzhou/2002		
	EF025110/Guangzhou/2006		
	FJ176779/Guangzhou/2006		
	FJ176780/Guangzhou/2006		
	FJ196843/Guangzhou/2006		
	FJ196844/Guangzhou8/2006		
	AY834999/Zhejiang/2004		
	DQ193572/Fujian/2004		
	EU280167/Guangzhou/2007		
	EU359008/Zhuhai/2007		
	FJ196841/Guangzhou/2003		
	FJ196842/Guangzhou/2003		
	FJ196845/Guangzhou/1991		
	FJ196847/Guangzhou/1997		
	JQ048541/Dongguan/2011		
DENV-2	AF350498/Guangdong/1980	63±6	
	AF119661/Hainan/1985		
	AF204177/Hainan/1989		
	AF204178/Guangxi/1987		
	AF276619/Fujian/2000		
	AF359579/Fujian/1999		
	EF051521/Zhongshan/2001		
	EU359009/Zhuhai/2007		
	FJ196851/Guangzhou/1998		
	FJ196852/Guangzhou/2001		
	FJ196853/Guangzhou/2003		
	FJ196854/Guangzhou/1993		
DENV-3	AF317645/Guangxi,	59±9	
	EU367962/China		
	GU189648/Zhejiang/2009		
	GU363549/Guangzhou/2009		
	JF504679/Zhejiang/2009		
	JN662391/Guangzhou/2009		
DENV-4	FJ196849/Guangzhou/1978,	82±9	
	FJ196850/Guangzhou/1990		
	JF741967/Guangzhou/2010		
	JQ822247/Zhejiang/2009		

**Table 3:** Comparison of the AA variations between the two serotypes in mainland China.

Dengue serotype	Group	Strains (accession number/geography/year)	AA variations in full-length (M±SD)	One-way ANOVA	AA variations of E (M±SD)^§^	AA variations of NS1(M±SD)^<$>\raster="rg1"<$>^
DENV-1	1	FJ196845/Guangzhou/1991	82.0±6.7	F = 3.9	–	–
		FJ196846/Guangzhou/1995		p = 0.024		
		EF032590/Guangzhou/1995				
		FJ196848/Guangzhou/1999				
		FJ196847/Guangzhou/1997				
	2	JN205310/Guangzhou/2002				
		FJ196841/Guangzhou/2003,	89.2±14.2		16.8±2.4	15.3±3.0
		FJ196842/Guangzhou/2003				
		AY835999/Zhejiang/2004				
		DQ193572/Fujian/2004				
		EF025110/Guangzhou/2006				
		FJ176779/Guangzhou/2006				
		FJ176780/Guangzhou/2006				
		FJ196843/Guangzhou/2006				
		FJ196844/Guangzhou/2006				
		EU280167/Guangzhou/2007				
		EU359008/Zhuhai/2007				
DENV-2	3	FJ196854/Guangzhou/1993	74.8±7.8		–	–
		FJ196851/Guangzhou/1998				
		AF359579/Fujian/1999				
		AF276619/Fujian/2000				
	4	EF051521/Zhongshan/2001	64±6.7		11±0.8	7.5±1.7
		FJ196852/Guangzhou/2001				
		FJ196853/Guangzhou/2003				
		EU359009/Zhuhai/2007				

Note: “§”: Mann-Whitney U test, Z = 2.96, p = 0.003; “<$>\raster="rg1"<$>”: Student’s t-test: t = 4.89, p<0.001; “−”: No statistics.

**Table 4:** AA variations in DENV-1 and DENV-2 between the Guangzhou city and other regions.

	DENV-1		Statistic	P value	DENV-2		Statistics	P value
	Guangzhou(14 strains)	Other regions(5 strains)			Guangzhou(4 strains)	Other regions(7 strains)		
NS1	–	–	–	–	10±3.7	5.7±1.6	t = 2.7	0.024
NS2B	–	–	–	–	3.3±1.0	1.7±0.8	t = 2.9	0.016
NS3	12.1±2.0	9.8±1.8	t = 2.3	0.034	12.8±1.5	8.4±4.5	Z = 2.2	0.042

Note: “−”: No statistics; “t”: using student-t test; “Z”: using Mann-Whitney test.

### Recombination Analysis

**Figure 4:**
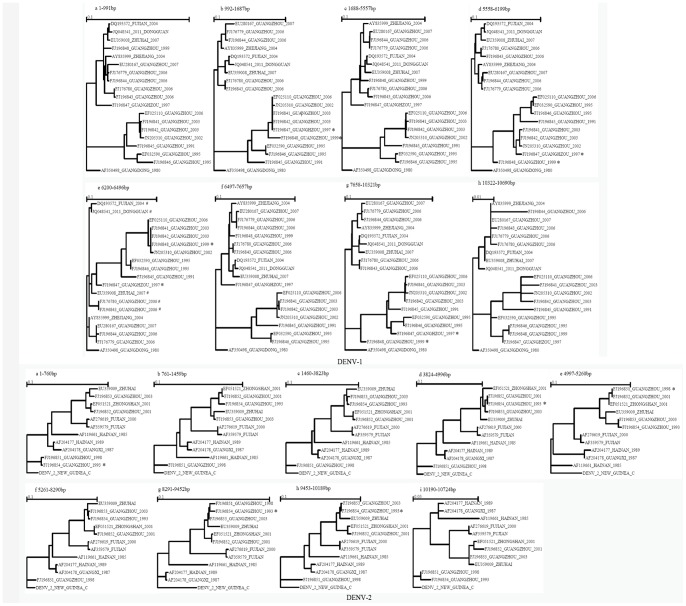
Phylogenetic analysis based on the breakpoints of DENV-1 and DENV-2 according to Datamonkey.

No recombination events were shown in the inter-serotype between DENV-1 and DENV-2, while significant recombination events were shown in the intra-serotype of DENV-1 and DENV-2. In DENV-1, seven breakpoints (location: 991, 1687, 5557, 6199, 6496, 7657 and 10321) were found, but only the first six breakpoints showed significant differences (P<0.01). Four breakpoints (location: 991, 1687, 5557 and 7657) occurred in the two isolates FJ196847 and FJ196848, while the remaining two breakpoints (location: 6199, 6496) were observed in the five isolates DQ193572, JQ048541, EU359008, FJ176780 and FJ196843 (Figure 4). In DENV-2, except for one non-significant breakpoint (location 1687), eight breakpoints (location: 760, 1459, 3823, 4996, 5260, 8290, 9452 and 10189) were observed. All of these breakpoints were found in the isolates FJ196851, FJ196852, FJ196853 and FJ196854 (Figure 4).

## Discussion

Phylogenetic analysis of the gene sequences obtained directly from patient sera provides a rapid approach for discriminating dengue viruses according to serotype, genotype and clade. This molecular epidemiological typing technique is widely used and accepted for the genotyping of dengue viruses. Thus far, nearly all phylogenetic analyses on DENV in mainland China have used the nucleotide sequence of the E gene to identify the genotypes for the DENV-1, DENV-2, DENV-3 and DENV-4 viruses [15], [16], and the E gene has not been evaluated to determine whether it is suitable for use in genotyping the virus. Additionally, spatial and temporal analyses were performed in previous epidemiological studies [27], [28], but few have been analyzed using the whole genome of the dengue virus.

In the present study, phylogenetic analyses were performed using the nucleotide sequences of the complete genome, the ORF and individual coding and non-coding genes from 19 DENV-1 and 11 DENV-2 isolates from mainland China. The results showed that the E gene would not improve the stratification of the different genotypes. Although the E gene has been thought to be effective in genotyping the dengue viruses, the topology and its overall evolutionary distance did not support its use in analyzing the evolution and selection pressure of dengue virus in mainland China (Figure 1 and Table 1). These results suggest that the viral evolution in the different genetic groups reflects differences in both the individual coding and non-coding genes. The topologies from these genes were not similar to the topology from the complete sequence. Additionally, this study showed that the ORF gene might be useful for genotyping and clade identification for the majority of the DENV-2 isolates analyzed. In the DENV-1 isolates, according to the topology from the complete sequence, the viruses could be classified into three genotypes and five clades, while one of the nodes was less than 90% certain according to the ORF gene (Figure 1: A, B). Thus, the ORF gene cannot be used to analyze the evolutionary distance and the selection pressure of the DENV-1 isolates from mainland China. These results greatly differ from a previously published study [14]. The reasons for these different results may be due to the different epidemic locations, the modes of evolution of the dengue viruses and different epidemic years. Additionally, this study had several limitations, including a small sample size. Therefore, if the aim of a study is to classify the dengue virus for the study of viral evolution and selection pressure, then there are no other target sequence(s) that can be selected apart from the complete sequences for the DENV-1 and DENV-2 isolates and the ORF gene for DENV-2 in mainland China.

Recently, the E gene region has been widely used for molecular characterization. However, the evolution of DENV involves not only in the E gene, but also other regions, particularly in the non-structural region, such as the NS1, NS2A, NS4B and NS5 regions [30], [31], [32]. In our study, there were significant differences not only in the AA variations of E gene region, but also in non-structural region. Therefore, phylogenetic analysis with the E gene region cannot replace the molecular characteristics of DENV in the study of viral evolution and selection pressure. Dengue has been epidemic in mainland China for 30 years. Mutations in regions other than the E gene may also influence the biological characteristics of DENV. Additionally, recombination events were observed in the E gene region of DENV-1 and DENV-2. Thus, the E gene might not be suitable for use in analyzing the molecular characteristics of the dengue virus in mainland China.

According to the number of AA variations in the four serotypes of DENV, the most variable AA sequence belonged to the DENV-1 serotype. This finding is consistent with the fact that the frequency of epidemics caused by the DENV-1 serotype is the highest [7], [33]. Interestingly more AA changes were observed in DENV-4 than in DENV-2 and DENV-3; however, the frequencies of DENV-2 (10 epidemics) and DENV-3 (8 epidemics) epidemics were more than that of DENV-4 (4 epidemics) [7], [33]. In other Southeast Asian countries, such as Thailand and Malaysia, the frequency of DENV-4 epidemic was also reported to be very low [34], [35], [36]. Thus, we conclude that a silent epidemic of DENV-4 may exist in Southeast Asian countries. However, few studies show the serotypes of the population with dengue in Southeast Asian countries. Therefore, a large epidemic survey is needed to prove this conclusion.

The AA changes of the DENV-1 isolates from 2001 to 2010 were more numoerous than those of the DENV-2 isolates from 2001 to 2010, and no significant difference was found between the DENV-1 and DENV-2 isolates from 1991 to 2000. Seven mutations were observed in the DENV-1 isolates, including the N290D, L402F and A473T mutations in the E gene region and the R101K, G105R, D340E and L349M mutations in the NS1 region, while no mutation was found in the DENV-2 in these locations (Figure 3). The E protein consists of three domains, designated as domains I (amino acids 1–51, 132–192 and 280–295), II (amino acids 52–131 and 193–279) and III (amino acid 296–393). In this study, one mutation occurred in domain I, which is an elongated domain, and the other two mutations were not found in these three domains. However, a recent research showed that the penultimate interaction, which involves the 402F residue, has hydrophobic contact with a conserved surface on domain II [37]. That study showed that it is important for DENV that the mutations occur in the E gene region. The NS1 protein plays a significant role in immune evasion during infection [38], [39]; thus, adaptive AA mutations may have occurred after 2001 to enhance the virus’ susceptibility to the human immune system. This may be one explanation for the high frequency of DENV-1 epidemics compared to that of DENV-2 after 2001.

The NS3 protein is a multifunctional enzyme with separate active sites involved in viral RNA replication and capping, including helicase, nucleoside 5′-triphosphatase (NTPase) and RNA 5′-triphosphatase (RNPase) activities [40]. Therefore, mutations in this gene could have significant effects on viral replication. In mainland China, there are significant differences in the mutations within the NS3 region between the Guangzhou isolates and the other regional isolates of DENV-1 and DENV-2. An increase in the AA mutation frequency of the Guangzhou isolates increased the survival opportunity of the virus, which may explain why dengue is mainly epidemic in Guangzhou compare to other sub-tropic areas of mainland China.

## Conclusion

According to this study, the complete sequence of DENV-1, as well as the complete genome or ORF sequence of DENV-2, is suitable or use in analyzing viral evolution and selection pressure, whereas the E genes are not. Additionally, the mutations in the E and NS1 regions may have had effects on the DENV-1 epidemics since 2001, and mutations in the NS3 region might affect the DENV-1 and DENV-2 epidemics in different regions.
